# Repurposing tricyclic drugs as cancer therapeutics: comparative analysis of antitumorigenic effects of chlorpromazine, amitriptyline and imipramine

**DOI:** 10.3389/fonc.2026.1827698

**Published:** 2026-07-02

**Authors:** Joos Berghausen, Eric Glasgow, Tinatin I. Brelidze

**Affiliations:** 1 Department of Pharmacology and Physiology, Georgetown University Medical Center, Washington, DC, United States; 2 Department of Oncology, Georgetown University Medical Center, Washington, DC, United States

**Keywords:** inhibitor, melanoma, neuroblastoma, triple-negative breast cancer, xenograft, zebrafish

## Abstract

**Introduction:**

Tricyclic drugs such as chlorpromazine (CPZ), amitriptyline (AmiT), and imipramine (ImiP) have demonstrated antitumorigenic potential, yet their relative potency and selectivity for different tumors remain unclear. Here, we conducted a comparative study of the antitumorigenic effects of these drugs on three major cancer types: breast cancer (MDA-MB-231), neuroblastoma (SH-SY5Y), and melanoma (A375).

**Methods:**

*In vitro* antitumorgenic effects were assessed using CellTiter-Blue viability assays and wound-healing assays, while *in vivo* effects were evaluated using zebrafish xenografts. Cancer cell growth inhibition and migration were examined across all three cancer cell lines following treatment with CPZ, AmiT, or ImiP.

**Results:**

In the *in vitro* experiments, inhibition of cancer cell growth was strongest for CPZ and weakest for ImiP, with AmiT showing intermediate effects across all three cell lines. In wound-healing assays, all three drugs significantly impaired migration in MDA-MB-231 and SH-SY5Y cells but had no effect on the migration of A375 cells. In the *in vivo* experiments, CPZ and AmiT significantly reduced tumor growth in MDA-MB-231 and SH-SY5Y xenografts, whereas ImiP inhibited tumor growth in a statistically significant manner only for MDA-MB-231 xenografts and did not inhibit tumor growth in SH-SY5Y xenografts. All three drugs failed to inhibit tumor growth in A375 xenografts.

**Discussion:**

Together, these findings demonstrate clear differences in antitumorigenic potency and cancer-type sensitivity among the tested tricyclic drugs, with CPZ being the most potent in both MDA-MB-231 and SH-SY5Y cells, followed by AmiT and then ImiP, in both *in vitro* and *in vivo* experiments. In contrast, all three drugs exhibited little or no antitumorigenic effect on A375 cells in both *in vitro* and *in vivo* experiments.. These findings further the efforts to repurpose the FDA-approved tricyclic drugs CPZ, AmiT, and ImiP for cancer treatment.

## Introduction

Drug repurposing is a frequently used drug discovery strategy that leverages the knowledge of the safety profile for already FDA-approved drugs for their use to treat a disease different than the prior indication. This approach could reduce time, cost, and risk associated with traditional drug discovery pipelines (1). Notable examples of successful drug repurposing include sildenafil, which was initially developed as an anti-hypertensive drug but subsequently approved for the treatment of erectile dysfunction (2), and thalidomide, which was initially used as an anti-nausea medication and subsequently repurposed as a cancer therapeutic for multiple myeloma (3).

**Figure 1:**
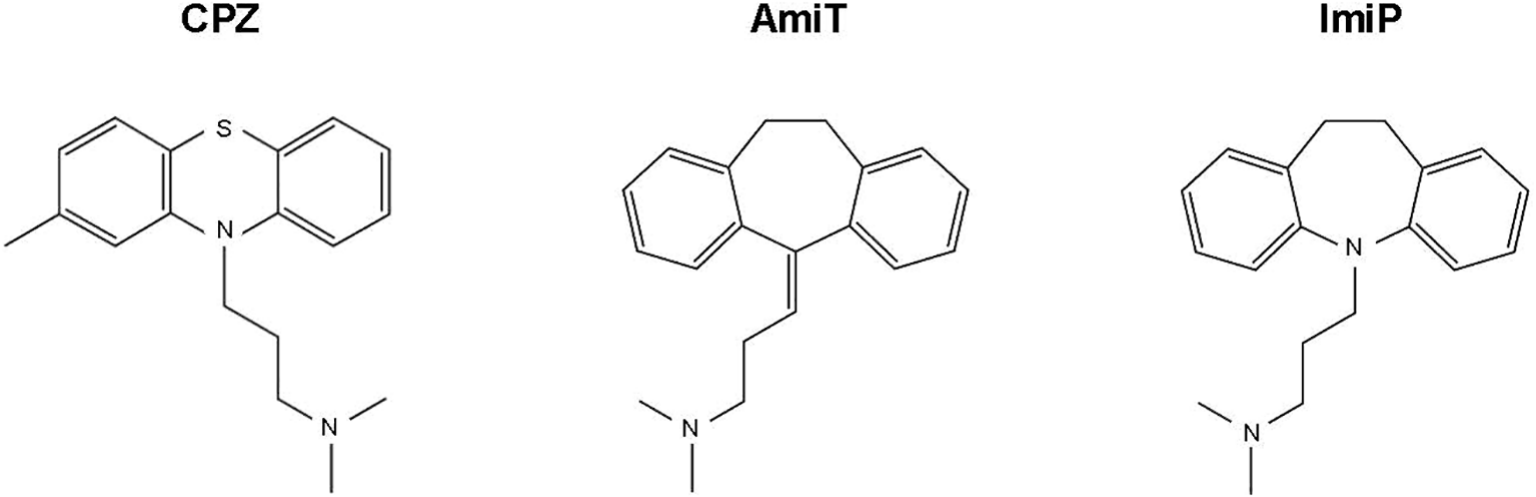
Chemical structures of the three tricyclic drugs CPZ, AmiT and ImiP.

Chlorpromazine (CPZ), amitriptyline (AmiT) and imipramine (ImiP) are FDA-approved tricyclic drugs that have been gaining interest in cancer therapy (**Figure 1**) (4). Chlorpromazine, a first-generation antipsychotic, is used to treat schizophrenia, bipolar disorder, and other psychotic disorders (5). While its mechanism of action is still not completely clear, it is thought to work through post-synaptic blockage of D2 receptors in the mesolimbic pathway (5). CPZ has been shown to affect breast cancer, colorectal cancer, brain tumors, skin cancer, leukemia, lymphoma, lung cancer, pancreatic cancer, and oral cancer in preclinical settings, as summarized by Kamgar-Dayhoff and Brelidze (4). Furthermore, an epidemiological study out of Denmark from 1957–1980 revealed that psychiatric patients who were treated with CPZ had a decreased risk of developing cancer, suggesting that CPZ at clinical doses could be anti-tumorigenic (6). AmiT and ImiP are tricyclic antidepressants that act primarily through inhibition of serotonin and norepinephrine reuptake (7). AmiT and ImiP show preclinical anti-tumorigenic effects on several cancer types, including melanoma, prostate cancer, lymphoma, lung cancer, colorectal cancer, breast cancer, and hepatocellular carcinoma (8). Importantly, depression is frequently observed in the oncology setting (9, 10). This substantial disease burden highlights the potential utility of tricyclic antidepressants in cancer care, as they may simultaneously address psychiatric comorbidities and cancer-related symptoms. Moreover, their established use in managing chemotherapy-induced neuropathic pain further underscores their therapeutic versatility (11, 12).

Although the antitumorigenic properties of CPZ, AmiT, and ImiP have been reported, the existing studies employ different methodologies, experimental models, and research objectives, complicating direct comparisons of the antitumorigenic properties of the three tricyclic drugs. For instance, the reported IC50 values for cell growth inhibition in the same cancer cell lines can differ three to four times (13). Therefore, to determine which of the tricyclic drugs holds the most promise for repurposing in cancer treatment, comparative studies utilizing the same protocols are needed. Here, we conducted a comparative study of antitumorigenic effects of CPZ, AmiT and ImiP to directly compare their therapeutic potential to inhibit cancer growth and migration of three major cancers: breast cancer (MDA-MB-231), neuroblastoma (SH-SY5Y) and melanoma (A375). Our *in vitro* cell viability experiments revealed that for all three cancer types CPZ was the most potent out of the three tricyclic drugs at inhibiting the cancer cell growth, followed by AmiT, whereas ImiP showed the smallest effect. CPZ demonstrated its strongest inhibition of cell growth in neuroblastoma cells and affected cell growth of the breast cancer and melanoma cells equally. AmiT showed the strongest cell growth inhibition for the breast cancer and neuroblastoma, affecting the growth of these two cell types equally. ImiP inhibited the growth of the three cell lines with almost the same potency, with slightly weaker effect on the growth of melanoma cells. Our *in vitro* wound healing experiments revealed that CPZ, AmiT, and ImiP have similar effects on the migration of neuroblastoma and breast cancer cells but failed to inhibit migration of melanoma cells. *In vivo* experiments using zebrafish xenografts revealed that each drug has significant tumor growth reduction for breast cancer cells, however, only CPZ and AmiT significantly reduced tumor growth for neuroblastoma cells. Interestingly, none of the drugs were able to decrease tumor growth in melanoma cells. Collectively, these data suggest that CPZ has the strongest antitumorigenic potential among the tested drugs, followed by AmiT and then ImiP, and that breast cancer and neuroblastoma are more susceptible to the tricyclic drugs than melanoma.

## Methods

### Cell culture

Stably GFP-expressing MDA-MB-231 (P20117), SH-SY5Y (P20103), and A375 (P20122) cells were purchased from Innoprot (Derio, Spain) and cultured according to the manufacturer’s protocol. Trypsin-free PBS-EDTA was used for cell detachment from the tissue culture dishes. Cells were cultured at 37 °C and 5% CO_2_.

### Preparation of drug solutions

Chlorpromazine hydrochloride (CPZ, Alfa Aesar, US), amitriptyline hydrochloride (AmiT, Sigma, US) and imipramine hydrochloride (ImiP, imipramine hydrochloride, Sigma, US) were dissolved in the cell culture media at a 1 mM concentration for CPZ and 2 mM concentrations for AmiT and ImiP. These solutions were then diluted to obtain the indicated concentrations using a serial dilution method. The stock solutions were prepared fresh before each experiment.

### Cell viability assay

For the cell viability assays, cells were seeded at a density of 10,000 cells per well in a 96-well plate. Following 24 hours of culture, cells were treated with CPZ, AmiT, and ImiP for 48 hours applied at up to 12 different concentrations, ranging from 0 µM to 1000 µM. Each concentration was applied in triplicate. The 48h treatment timeline has been previously determined to be the optimal duration, as it allows for maximal drug exposure time, yet the cells do not reach confluency in the control group (14, 15). CellTiter-Blue assay (Promega, US) was used to determine cell viability. The CellTiter-Blue assay is based on the conversion of non-fluorescent resazurin into fluorescent resorufin by living cells. The resazurin fluorescence upon excitation at 560 nm was recorded at 590 nm with the Synergy H4 Plate Reader (BioTek Instruments, US). The experiments were repeated three times. For each cell line, the fluorescence signal obtained over the range of applied drug concentrations was normalized to its corresponding control group (untreated) and fitted with the Hill equation to calculate IC_50_ values.

Comparison between IC_50_ values was performed using one-way ANOVA. P < 0.05 was considered statistically significant.

### Wound healing assay

For the wound healing assay, the cancer cells were seeded at a density of 50,000 cells per well into 24-well plates. Once the wells reached 80%-90% confluency, a wound was introduced by scratching across the bottom of the well with a 200 μL pipette tip. Following the wound introduction, the wells were washed three times with PBS to remove the debris and were treated with either media alone (control group) or media containing 4 μM CPZ, 10 μM AmiT, or 20 μM ImiP (treatment group). These concentrations for the wound healing experiments were selected because our cell viability experiments indicate that they do not affect cell growth. For each well, three representative images were collected along the wound at 0 and 48 hours after the wound introduction using an Olympus IX-71 (Olympus, Japan) inverted microscope. The wound size was calculated for each day using the ImageJ Wound Healing Size Tool plugin (16). The experiments were repeated three times. Changes from 0 to 48 hours were calculated for each well individually, and a comparison between the treatment and control groups was performed using two-way ANOVA. P < 0.05 was considered statistically significant.

### Zebrafish xenografts

All animal procedures were conducted in accordance with NIH guidelines for the care and use of laboratory animals and approved by the Georgetown University Institutional Animal Care and Use Committee (Protocol 2017-0078).

For the zebrafish xenograft experiments, 100–200 cancer cells were injected into the yolk-sac of 2 days post-fertilization (dpf) zebrafish embryos, as described before (17, 18). The zebrafish line *Tg(kdrl:mCherry)^y206^* that expresses fluorescent protein mCherry in the endothelial cells, was used. Around 200 embryos were divided into treatment and control groups. The animals in the treatment group were treated daily with CPZ, AmiT, or ImiP added to the fish water at the maximal tolerable dose (MTD) of 5 µM, 15 µM, and 35 µM, respectively. For the MTD assay, 2 dpf zebrafish larvae were placed in 1 mL fish water solution with increasing concentrations of drug, starting at 5 µM and increasing in 5 µM increments. Five fish were placed per well in a 24-well plate. The larvae were monitored over 5 days. The highest concentration at which no mortality and minimal effects on zebrafish development and general behavior were observed was designated as the MTD (19). Changes in tumor sizes were monitored daily for four days post-injection using an Olympus IX-71 inverted microscope, as previously described (18). Tumor sizes were calculated using ImageJ by measuring tumor area (17). Animals lacking measurable tumors were excluded from the study. Changes from day 0 to day 4 were calculated for each individual fish, and comparisons between treatment and control groups were performed using One-way ANOVA. P < 0.05 was considered statistically significant. Sex of zebrafish embryos becomes clear only at the age of 3 months, well after the first week, to which we limit our experiments. Therefore, sex as a biological variable does not need to be considered for the xenograft experiments described in this study.

## Results

### CPZ demonstrates the strongest potency at inhibiting cell growth of MDA-MB-231, SH-SY5Y or A375 cells *in vitro*, followed by AmiT and ImiP

**Figure 2:**
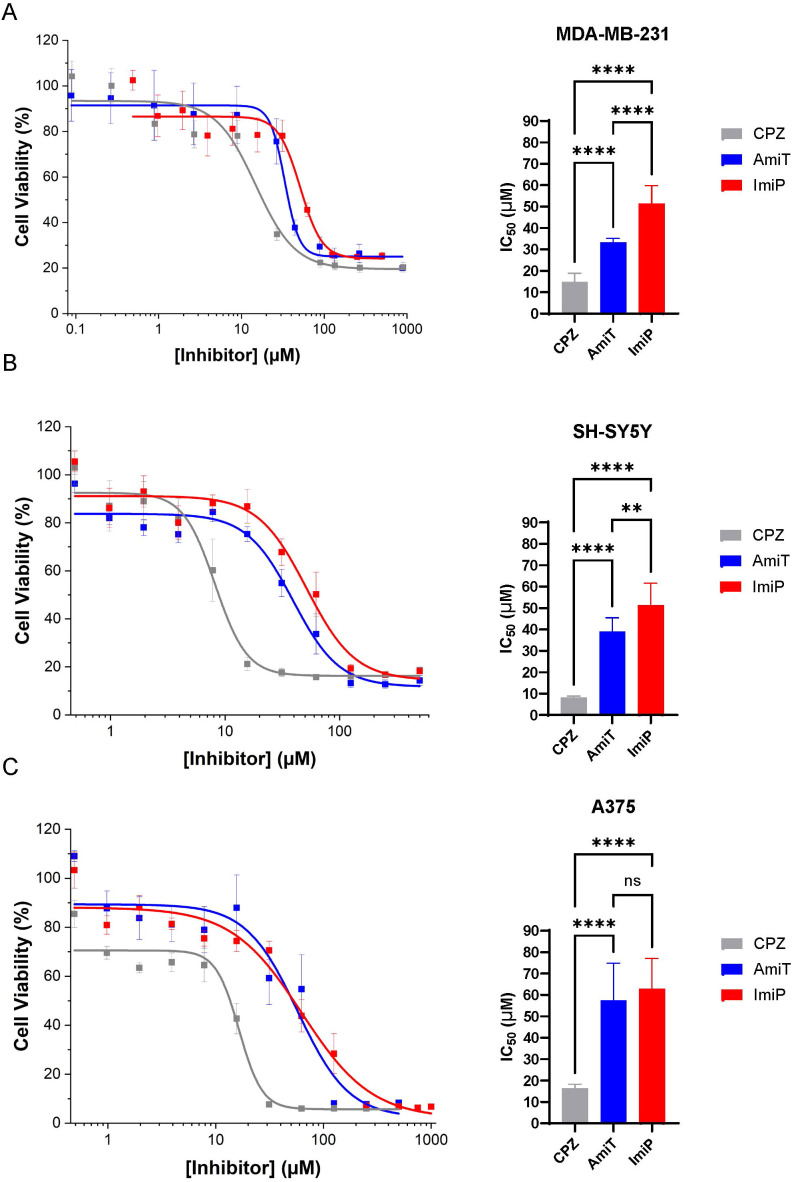
CPZ, AmiT, and ImiP inhibit MDA-MB-231, SH-SY5Y and A375 cancer cell growth in a concentration-dependent manner. Viability of MDA-MB-231 (A, left), SH-SY5Y (B, left) and A375 (C, left) cells over the range of CPZ (grey), AmiT (blue) or ImiP (red) concentrations. The lines represent fits of the data with the Hill equation, with the IC_50_ values described in the text. Each data point is an average of three technical replicates for three different biological samples. Bar graphs of averaged IC_50_ values for CPZ, AmiT and ImiP for MDA-MB-231 (A, right), SH-SY5Y (B, right) and A375 (C, right) cells. ANOVA analysis was used to determine statistical significance (****P < 0.0001; ***P < 0.001; **P < 0.01; *P <0.05). The data are presented as mean ± SD throughout the figure.

Previous studies have demonstrated the antitumorigenic effect of the tricyclic drugs CPZ, AmiT, and ImiP in various cancer models (4, 8). However, differences in experimental conditions and assays used in the studies complicate direct comparison of the relative potency and cancer type selectivity of the tricyclic drugs. To directly compare the potency and cancer type selectivity of the tricyclic drugs, we investigated the effect of CPZ, AmiT, and ImiP on the growth of MDA-MB-231, SH-SY5Y, and A375 cells using the CellTiter-Blue cell viability assay (Promega). The results for AmiT are from our previous publication (20). CPZ caused the strongest concentration-dependent inhibition of MDA-MB-231 cell growth with the IC_50_ value of 14.87 ± 3.99 μM, followed by AmiT with the IC_50_ value of 33.24 ± 1.79 μM, and ImiP with the IC_50_ value of 51.40 ± 8.42 μM (**Figure 2A**). A similar potency pattern was observed for SH-SY5Y cell growth, with the strongest effect caused by CPZ with the IC_50_ value of 8.18 ± 0.75 μM, followed by AmiT with the IC_50_ value of 39.11 ± 6.41 μM, and the smallest effect seen was caused by ImiP, with the IC_50_ value of 51.36 ± 10.25 μM (**Figure 2B**). Finally, CPZ induced the strongest inhibition of A375 cell growth with an IC_50_ value of 16.45 ± 1.81 μM, while AmiT and ImiP inhibited A375 cell growth with statistically similar potency with the IC_50_ values of 57.38 ± 17.46 μM and 62.88 ± 14.17 μM, respectively (**Figure 2C**).

**Figure 3:**
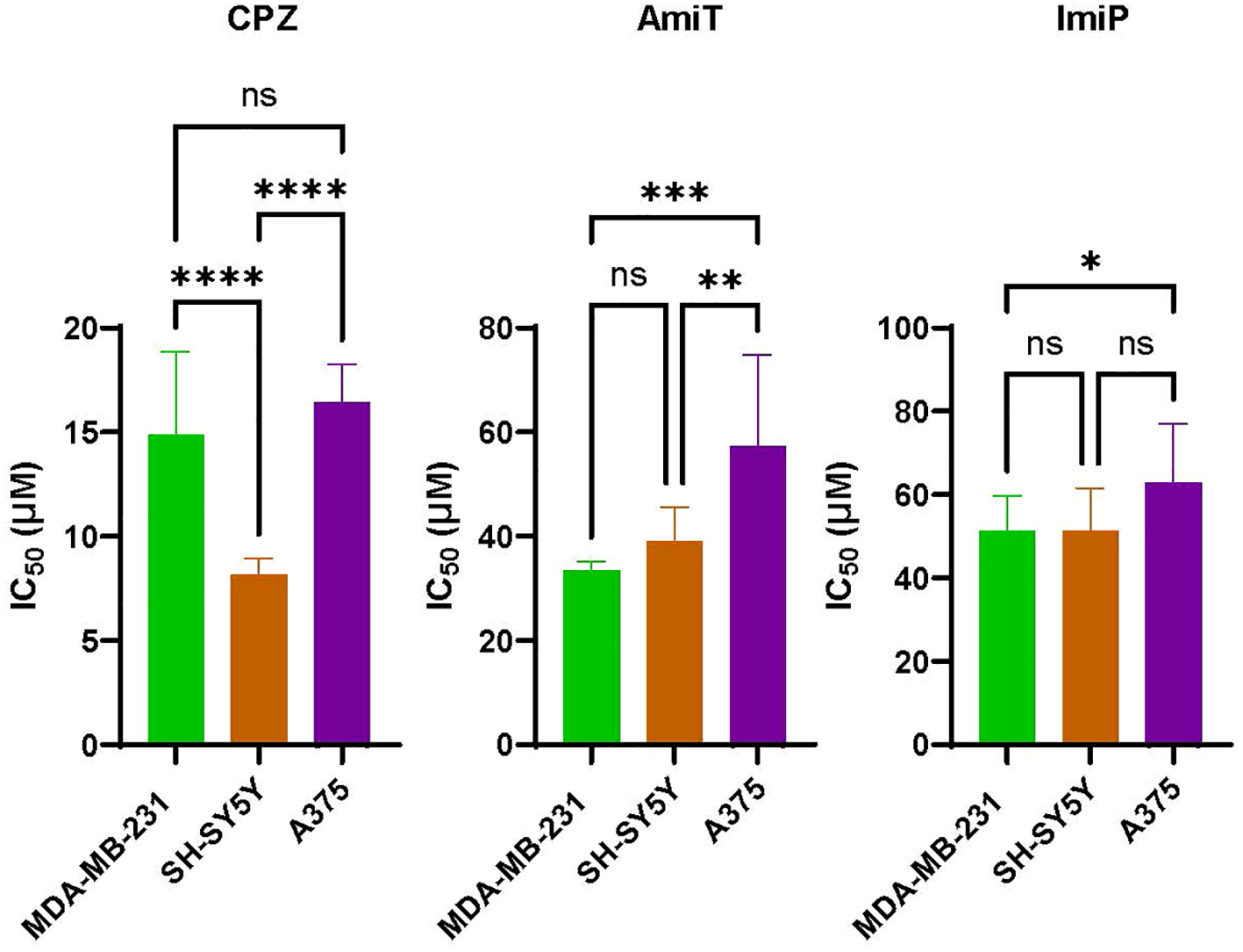
CPZ has the strongest inhibitory effect on the tested cancer cell growth among the three tricyclic drugs. Bar graphs of averaged IC_50_ values for CPZ (left), AmiT (middle) and ImiP (right) for MDA-MB-231 cells (green), SH-SY5Y cells (brown), and A375 cells (purple). ANOVA analysis was used to determine statistical significance (****P < 0.0001; ***P < 0.001; **P < 0.01 *P <0.05). The data are presented as mean ± SD throughout the figure.

Overall, CPZ exhibited the strongest inhibition of cell growth across all cell lines tested, followed by AmiT and then ImiP (**Figure 3**). CPZ exhibited the greatest potency against SH-SY5Y cells, while producing comparable effects on the cell growth inhibition in MDA-MB-231 and A375 cells (**Figure 3**, left). AmiT inhibited the growth of MDA-MB-231 and SH-SY5Y cells with the same potency but had a markedly weaker effect on A375 cell growth (**Figure 3**, middle). In contrast, ImiP caused similar inhibition of cell growth for all three tested cell lines with a slightly higher but statistically significant sensitivity in MDA-MB-231 cells relative to SHSY5Y cells (**Figure 3**, right). Notably, AmiT and ImiP showed comparable levels of cell growth inhibition in A375 cells. Taken together, these results indicate that CPZ could provide the greatest therapeutic benefit in neuroblastoma (SH-SY5Y), whereas melanoma (A375) appears less responsive to all three tested tricyclic drugs. Noteworthy, the effect on the cell viability seems to saturate at ~20% for MDA-MB-231 and SH-SY5Y cells for all three tricyclic drugs (**Figures 2A, B**), while the effect on A375 cell viability saturates at ~5% for all three tricyclics tested (**Figure 2C**). This suggests that, although, the tricyclic drugs are more potent at inhibiting cell growth of MDA-MBA-231 and SH-SY5Y cells, the efficacy of the cell growth inhibition is higher in A375 cells.

### CPZ, AmiT, and ImiP inhibit cell migration *in vitro* for MDA-MB-231 and SH-SY5Y but not A375 cells

**Figure 4:**
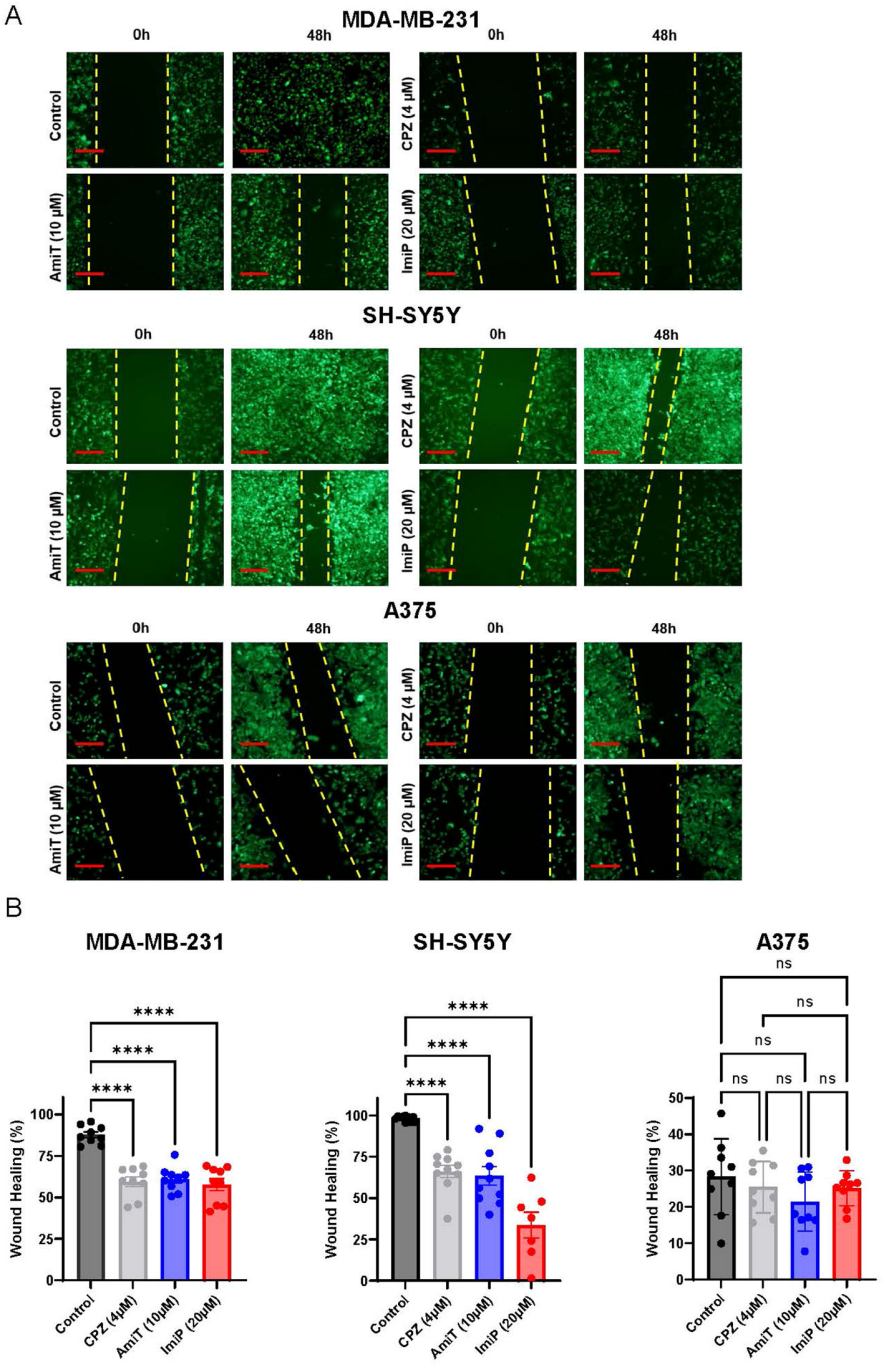
CPZ, AmiT, and ImiP inhibit *in vitro* migration of MDA-MB-231 and SH-SY5Y but not A375 cells. **(A)** Representative images of the wound healing of MDA-MB-231 (top), SH-SY5Y (middle), and A375 (bottom) cells, untreated or treated with 4 µM CPZ, 10 µM AmiT or 20 µM ImiP at 0 and 48 hours after scratch. Scale bar = 0.25 mm. **(B)** Bar graph summary of the % wound healing in the absence (black) and presence of CPZ (grey), AmiT (blue), and ImiP (red) at 48 hours post-scratch for MDA-MB-231 (left), SH-SY5Y (middle), and A375 (right) cells. Statistical significance was determined with ANOVA test (****P < 0.0001). The data are presented as mean ± SD and are averages of three technical replicates for three different biological samples for each tested condition.

Metastasis is the primary reason for cancer-related deaths (21). Therefore, to fully characterize a drug’s anti-tumorigenic potential, the analysis of its ability to inhibit migration, a critical early step in metastasis, is of utmost importance. To investigate the effect of the tricyclic drugs on cancer cell migration, we employed a commonly used wound healing assay, where a scratch wound healing was analyzed in the absence and presence of 4μM CPZ, 10 μM AmiT and 20μM ImiP at 0 and 48 hours after the wound introduction (**Figure 4**). The results for AmiT are from our previous publication (20). In the absence of any drug, the wound was almost entirely healed at 48 hours post scratch for MDA-MB-231 and SH-SY5Y cells, and only 28.29 ± 10.4% healed for A375 cells (**Figures 4A, B**). Treatment with CPZ, AmiT, or ImiP significantly impaired cell migration in MDA-MB-231 and SH-SY5Y cells 48 hours post scratch (**Figure 4B**, left and middle). Specifically, CPZ-treated cells showed 59.41 ± 9.00% and 66.18 ± 11.60% wound healing, AmiT-treated cells showed 61.03 ± 7.5% and 63.42 ± 17.9% wound healing and ImiP-treated cells displayed 57.69 ± 11.10% and 33.72 ± 20.60% wound closure for MDA-MB-231 and SH-SY5Y, respectively (**Figure 4B**, left and middle). Interestingly, none of the drugs produced a statistically significant reduction in wound healing in A375 cells at 48 hours post-scratch (**Figure 4B**, right). To test whether the effect of the drugs on the A375 cell migration requires a longer time period to manifest, we monitored wound healing in A375 cells for a longer time. Even at 120 hours post-scratch, the wound healing was incomplete (66.39 ± 9.4%) in the absence of any drugs (**Supplementary Figure 1A**). Importantly, the wound healing was not statistically different in the presence of CPZ, AmiT or ImiP, with 63.21 ± 9.29%, 62.46 ± 6.0%, and 56.01 ± 7.96% wound healed, respectively (**Supplementary Figure 1B**). Taken together, these findings indicate that all drugs inhibited the migration of MDA-MB-231 and SH-SY5Y cells, with ImiP exerting the most pronounced anti-migratory effect in SH-SY5Y. In contrast, none of the drugs significantly affected migration in A375 cells.

### CPZ and AmiT decrease tumor growth *in vivo* for SH-SY5Y and MDA-MB-231 but not A375 cells

**Figure 5:**
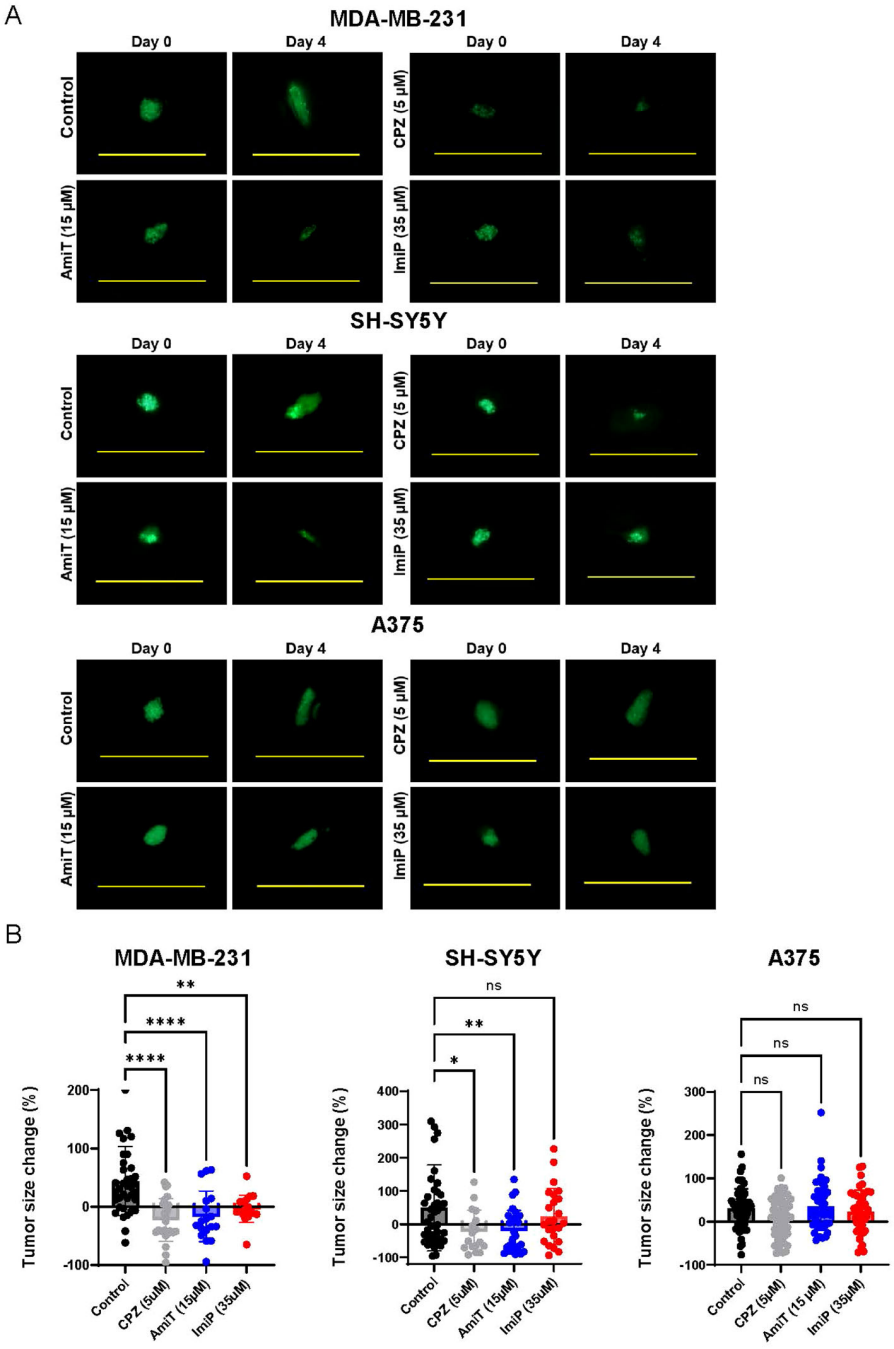
CPZ and AmiT decrease tumor growth *in vivo* for SH-SY5Y and MDA-MB-231 but not A375 cells. **(A)** Representative images of zebrafish xenografts with GFP-expressing MDA-MB-231 (top), SH-SY5Y (middle), and A375 (bottom) cells, untreated or treated with 5 µM CPZ, 15 µM AmiT or 35 µM ImiP, as indicated, at day 0 and day 4 after the cell injection. Scale = 1mm. **(B)** Quantification of % changes in tumor size on Day 4 after the implantation of MDA-MB-231 (left), SH-SY5Y (middle), and A375 (right) xenografts, as indicated, untreated (black) or treated with 5 µM of CPZ (grey), 15 µM AmiT (blue) or 35 µM of ImiP (red). The number of zebrafish used for MDA-MB-231 xenografts was: N = 37 for the untreated group and N = 24 for CPZ, N = 18 for AmiT, and N = 18 for ImiP treatment groups. The number of zebrafish used for SH-SY5Y xenografts was: N = 42 for the untreated group and N = 17 for CPZ, N = 31 for AmiT, and N = 24 for ImiP treatment groups. The number of zebrafish used for A375 xenograft was: N = 58 for the untreated group and N = 52 for CPZ, N = 47 for AmiT, and N = 42 for ImiP treatment groups. ANOVA analysis was used to determine statistical significance (****P < 0.0001; **P < 0.01 *P <0.05). The data are presented as mean ± SD throughout the figure.

Our experiments indicate that tricyclic drugs decrease cancer growth in a concentration-dependent manner in cell culture experiments. To investigate whether the anti-proliferative effects seen *in vitro* are also replicable *in vivo*, we employed zebrafish xenografts. Prior to xenografts, for each drug we conducted experiments to identify the maximal tolerable dose (MTD), a highest drug concentration that showed no detectable effect on zebrafish mortality, development, and general behavior. The MTD for CPZ was determined to be 5μM, for AmiT 15μM, and for ImiP 35μM. For the xenograft experiments, 2 dpf zebrafish larvae were implanted with MDA-MB-231, SH-SY5Y, and A375 cells constitutively expressing GFP, as we described before (17). The larvae were either left untreated or treated with the tricyclic drugs added to the fish water. Tumor size was determined immediately post-implantation (Day 0) and 96 hours post-implantation (Day 4) (**Figure 5A**). After four days of treatment, CPZ and AmiT significantly decreased tumor size in larvae engrafted with MDA-MB-231 and SH-SY5Y cells compared to their controls, while ImiP only showed a significant decrease in tumor size for MDA-MB-231 engrafted zebrafish (**Figure 5B**, left and middle). None of the drugs showed a statistically significant decrease in tumor size for zebrafish engrafted with A375 compared to controls (**Figure 5B**, right). In MDA-MB-231 xenografts, tumor size increased by 44.11% by Day 4 in the untreated (control) group, and decreased by 22.42%, 16.32% and only by 3.46% in the CPZ-, AmiT- and ImiP-treated groups, respectively (**Figure 5B**, left). In SH-SY5Y xenografts, tumor size increased by 48.94% in the untreated group, and decreased by 22.55% and 20.02% in the CPZ- and AmiT-treated groups, respectively (**Figure 5B**, middle). Interestingly, ImiP treatment resulted in a 23.76% increase on Day 4, which was statistically similar to the increase observed in the untreated group (**Figure 5B**, middle). In A375 xenografts, tumor size increased by 30.29% by Day 4 in the untreated group, and increased by 8.2%, 34.86% and 22.85% in the CPZ-, AmiT- and ImiP-treated groups, respectively (**Figure 5B**, right). Overall, only MDA-MB-231 xenografts were susceptible to treatment with all three tricyclic drugs, and only CPZ and AmiT were able to decrease tumor growth in SH-SY5Y xenografts. Interestingly, none of the drugs were able to inhibit tumor growth in a statistically significant manner for A375 xenografts. This mirrors our *in vitro* results, where the effect of the tricyclic drugs on the growth of A375 cells was much weaker than for MDA-MB-231 and SH-SY5Y cells.

## Discussion

In this study, we performed a comparative analysis of the antitumorigenic effects of three FDA-approved drugs, chlorpromazine (CPZ), amitriptyline (AmiT), and imipramine (ImiP), against breast cancer (MDA-MB-231), neuroblastoma (SH-SY5Y), and melanoma (A375) in both *in vitro* and *in vivo* models. All three drugs reduced cell viability in a concentration-dependent manner, with CPZ exhibiting the strongest effect on cell growth across the three tested cell lines, followed by AmiT and then ImiP. The potency for cell growth inhibition was strongest in SH-SY5Y cells and weakest in A375 cells for all three drugs, while the efficacy of cell growth inhibition was higher for A375 cells than for MDA-MB-231 and SH-SY5Y cells. In wound-healing assays, all three drugs significantly impaired the migration of MDA-MB-231 and SH-SY5Y cells, however, no antimigratory effects were observed in A375 cells. Consistent with the *in vitro* results, zebrafish xenografts revealed that CPZ and AmiT significantly reduced tumor size in MDA-MB-231 and SH-SY5Y xenografts, while ImiP exhibited statistically significant reduction of the grafted tumor only in MDA-MB-231 xenografts. Notably, no significant growth suppression was observed in A375 xenografts for any of the three drugs. Collectively, these findings suggest that CPZ, followed by AmiT, has the strongest anticancer potential among the three FDA-approved drugs tested, and that neuroblastoma and breast tumors will be responsive to the treatment with the tricyclic drugs.

Previous reports described antitumorigenic properties of CPZ, AmiT, and ImiP across diverse cancer models. CPZ has been shown to suppress multiple malignancies, including breast, colorectal, and brain cancers, in a preclinical setting (4). Reported IC_50_ values for CPZ, AmiT and ImiP are summarized in **Table 1**. The IC_50_ values for the inhibition of cancer growth by CPZ include 3.7 μM for colorectal cancer cells (22), 27.02 μM for MCF-7 breast cancer cells (23), 26.65 and 23.49 μM for oral cancer cells (24), and 4.5 μM for medulloblastoma (25). These IC_50_ values were determined using different protocols and assays, including MTT, MTS, and cancer sphere assays. This makes direct comparisons between cancer types nearly impossible, highlighting the need for standardized comparative studies. AmiT and ImiP have similarly demonstrated preclinical antitumorigenic activity in various malignancies, including melanoma, lung cancer, prostate cancer, lymphoma, multiple myeloma, and breast cancer (8). A recent publication showed that AmiT was able to inhibit cell growth of neuroblastoma (SH-SY5Y) cells with an IC_50_ value of 59.78 μM (26), which is slightly higher than the IC_50_ value of 39.11 μM for SH-SY5Y, reported here. This small variance in IC_50_ value could be due to procedural differences, such as using MTT assay instead of CellTiterBlue, or differences in incubation times (one hour instead of two), demonstrating the need for comparative studies. AmiT also decreased growth of two melanoma cell lines (SKMEL28, and SKMEL2) with the IC50 values of 43 μM and 35 μM, respectively (27). Another report showed that AmiT was able to reduce cell proliferation of colon cancer when treated at 50 μM AmiT concentration (28). ImiP has been shown to inhibit the growth of glioma cells, with the IC_50_ values ranging from 13.3 μM to 345.6 μM for six different glioma cell lines tested (29). It has also been reported that ImiP inhibits the growth of neuroblastoma cells (SH-SY5Y) with an IC_50_ value of 223.3 μM (30). This is substantially higher than the IC_50_ of 51.36 μM reported here, further highlighting the need for standardized comparative studies. Preclinical studies also show that the tricyclic drugs have anti-metastatic effects. For instance, *in vitro* studies show that CPZ inhibits the migration of mouse colon carcinoma cells (31) and pancreatic cancer cells (32), both at 10 μM concentrations. ImiP has been shown to inhibit the migration and invasion of prostate cancer cells at 12.5 μM (33) and bladder cancer cells at 30 μM (34). Additionally, ImiP was able to inhibit the migration of breast cancer cells (MDA-MB-231) at 20 μM concentrations (35), which is in agreement with the results reported in this study. Even though the concentrations used to test the anti-migratory effects of the tricyclic drugs do not differ as much as the reported IC_50_ values for the cancer cell growth inhibitions, the migration assessment in these studies was conducted using different protocols, making the direct comparison of the results challenging. Nevertheless, the IC_50_ values for cancer cell inhibition and concentrations that show anti-metastatic effects are mostly in the mid-micromolar range. CPZ, AmiT, and ImiP have been shown to have clinically observed plasma concentrations in the low micromolar range (0.1 μM – 2 μM) (36–40). However, it has been well established that tricyclic drugs can accumulate in tissues, and with long-term administration to the patients, the effective cellular concentrations are expected to be 10 to 1000-fold higher (41–43). Therefore, the concentrations used in the pre-clinical studies are clinically relevant. Additionally, many effective clinically used cancer therapeutics have relatively high IC_50_ values in cell culture experiments. For instance, cisplatin used to treat different solid tumors, including lung, ovarian and testicular cancers, inhibits A549 human lung cancer cell growth with the IC_50_ ranging from ~30 to ~70 μM 48 hours after the drug application (13). This further indicates that it is difficult to predict drugs’ clinical effectiveness solely based on the IC50 values determined from the cell culture experiments. A broader preclinical and clinical studies are needed to fully evaluate therapeutic potential of a promising antitumorigenic agents. Further investigating the effects of the tricyclic drugs on non-malignant breast, neuronal and epithelial cells would be valuable for assessing potential toxicity. However, their status as FDA-approved drugs with well-established safety profiles mitigates these concerns, provided their antitumorigenic effects are achieved at clinically acceptable doses.

**Table 1:** Summary of IC_50_ values for CPZ, AmiT and ImiP in different cell lines from the current study and previously published studies.

Drug	Cell line	IC50 (µM)	Experimental design	References
CPZ	MDA-MB-231 (breast cancer)	14.87 ± 3.99	• 48 h after treatment • CellTiter-Blue	Current study
	SH-SY5Y (neuroblastoma)	8.18 ± 0.75	• 48 h after treatment • CellTiter-Blue	Current study
	A375 (melanoma)	16.45 ± 1.81	• 48 h after treatment • CellTiter-Blue	Current study
	HCT116 (colorectal cancer)	3.7	• 48 h after treatment • MTT assay	(19)
	MCF-7 (breast cancer)	27.02	• 24 h after treatment • MTS assay	(20)
	HSC-3 (oral cancer)	26.65 ± 1.1	• 24 h after treatment • MTT assay	(21)
	Ca9-22 (oral cancer)	23.49 ± 1.26	• 24 h after treatment • MTT assay	(21)
	DAOY (medulloblastoma)	4.5	• Cancer sphere size	(22)
AmiT	MDA-MB-231 (breast cancer)	33.24 ± 1.79	• 48 h after treatment • CellTiter-Blue	Current study
	SH-SY5Y (neuroblastoma)	39.11 ± 6.41	• 48 h after treatment • CellTiter-Blue	Current study
	A375 (melanoma)	57.38 ± 17.46	• 48 h after treatment • CellTiter-Blue	Current study
	SKMEL28 (melanoma)	43	• 6 days • ATP-TCA	(24)
	SKMEL2 (melanoma)	35	• 6 days • ATP-TCA	(24)
	SH-SY5Y (neuroblastoma)	59.78 ± 2	• 48 h after treatment • MTT	(23)
ImiP	MDA-MB-231 (breast cancer)	51.40 ± 8.42	• 48 h after treatment • CellTiter-Blue	Current study
	SH-SY5Y (neuroblastoma)	51.36 ± 10.25	• 48 h after treatment • CellTiter-Blue	Current study
	A375 (melanoma)	62.88 ± 14.17	• 48 h after treatment • CellTiter-Blue	Current study
	U87 (glioma)	13.3 ± 1.1	• 48 h after treatment • CCK8	(25)
	U251 (glioma)	24 ± 1.1	• 48 h after treatment • CCK8	(25)
	U373 (glioma)	345.6 ± 1.4	• 48 h after treatment • CCK8	(25)
	LN229 (glioma)	152.7 ± 1.0	• 48 h after treatment • CCK8	(25)
	GL261 (glioma)	43.8 ± 1.0	• 48 h after treatment • CCK8	(25)
	GBM (primary glioma)	22.1 ± 1.1	• 48 h after treatment • CCK8	(25)
	SH-SY5Y (neuroblastoma)	223.3	• 48 h after treatment • MTT	(27)

Consistent with the clinical relevance of the reported anti-tumorigenic effects of the tricyclic drugs, epidemiological studies in psychiatric patients have associated CPZ, AmiT, ImiP treatment with reduced cancer risk, further underscoring the translational promise of repurposing these drugs for cancer treatment. A Danish epidemiological study demonstrated a decreased risk of developing cancer when psychiatric patients were treated with CPZ (6). Additionally, a case report demonstrated that a patient treatment with CPZ led to tumor regression of squamous-cell carcinoma of the larynx (44). Furthermore, cumulative high-dose treatment with CPZ led to decreased incidence rate of prostate cancer in a case-control study of 6168 schizophrenic patients (45). The use of AmiT and ImiP has also been associated with a decreased risk of developing certain cancers, including oral cancer (46), colorectal cancer, glioma (47), and gynecological cancers, such as cervical, ovarian, and uterine cancers (48). However, some reports show that tricyclic drugs could also increase the risk for certain cancers, including breast cancer (49) and lung cancer (50). These studies do not separate patients taking specific tricyclic drugs. As our study demonstrates, there are substantial differences in the anti-tumorigenic effects of different tricyclics. Therefore, it is possible that the increased cancer risk in the population studies does not reflect on the anti-tumorigenic potential of CPZ, AmiT and ImiP. More controlled population studies, on larger cohort of psychiatric patients would be beneficial for further assessing the clinical potential of repurposing CPZ, AmiT and ImiP for cancer treatment. AmiT and ImiP are especially attractive as candidates for drug repurposing, given the high prevalence of depression in oncology patients. For example, a 2018 cross-sectional study of cervical cancer patients in Thailand reported a 13.5% prevalence of depression, far exceeding national estimates of 0.3–2.4% (9). Likewise, a German cohort study of 4,020 individuals with cancer identified depressive symptoms in roughly 25% of patients, which represents a five-fold increase compared to the national average (10). Since AmiT and ImiP have been also used to treat chemotherapy-induced neuropathic pain (11, 12), repurposing these drugs for cancer treatment would have added benefits for pain management and depression reduction in cancer patients.

Our *in vitro* experiments show that CPZ exhibits the strongest inhibition of cancer cell growth across all three tested cancer types, followed by AmiT and then ImiP. The breast cancer and neuroblastoma were more susceptible to the treatment with the tricyclic drugs than melanoma. Our *in vivo* experiments were in agreement with the findings of the *in vitro* experiments. Indeed, in zebrafish xenografts, CPZ and AmiT showed a significant inhibition of tumor growth in breast cancer and neuroblastoma xenografts, with ImiP inducing a weak but still statistically significant reduction of tumor growth in breast cancer xenografts and no significant reduction in neuroblastoma xenografts. Tumor growth in A375 xenografts was not affected by any of the three tricyclic drugs. Taken together, CPZ and AmiT were the most promising candidates for inhibiting breast cancer and neuroblastoma growth, whereas ImiP demonstrates a weaker effect, especially in the *in vivo* experiments. It seems that melanoma growth is the least responsive to the tricyclic drugs out of the three tumor types tested, with no significant reduction observed in the *in vivo* experiments. In contrast to the effect on the cancer cell growth, ImiP was the most effective out of the three tested drugs in suppressing the migration of cancer cells. ImiP inhibited the migration of the breast cancer cells at the same level as CPZ and AmiT and showed a stronger inhibition of the cell migration for neuroblastoma cells relative to CPZ and AmiT. The equal or enhanced inhibition of migration by ImiP in SH-SY5Y relative to CPZ and AmiT is intriguing, given ImiP’s weaker effect on cell growth compared to CPZ and AmiT. This divergence could suggest that the anti-migratory ability occurs through mechanisms distinct from those governing cell growth. Interestingly, melanoma cells were consistently unaffected by all three tricyclic drugs in our *in vitro* migration experiments and *in vivo* xenografts. Although prior studies have reported anti-melanoma effects for AmiT, such outcomes may depend on experimental context, including drug concentrations, treatment duration, and specific assays used (27).

Taken together, this study provides a direct comparison of antitumorigenic properties of CPZ, AmiT, and ImiP for breast cancer, neuroblastoma, and melanoma studied under the same experimental conditions and with the same methodologies. Our results strongly support further exploration of repurposing CPZ and AmiT for the treatment of breast cancer and neuroblastoma growth, and ImiP for its therapeutic potential for inhibiting migration in breast cancer and neuroblastoma.

## Data Availability

The original contributions presented in the study are included in the article/**Supplementary Material**. Further inquiries can be directed to the corresponding author.

## Supplementary material

Supplementary Figure 1 CPZ, AmiT, and ImiP do not affect cell migration in A375 cells. (A) Representative images of the wound healing of A375 cells, untreated or treated with 4 µM CPZ, 10 µM AmiT or 20 µM at 0 and 120 hours after scratch. Scale bar = 0.25 mm. (B) Bar graph summary of the % wound healing in the absence (black) and presence of 4 µM CPZ (grey), 10 µM AmiT (blue) or 20 µM (red) at 120 hours post-scratch for A375 cells. Statistical significance was determined with ANOVA test (ns P > 0.05). The data are presented as mean ± SD and are averages of three technical replicates for three different biological samples for each tested condition.

## References

[1] Albuquerque PC Zicker F Fonseca BP . Advancing drug repurposing research: Trends, collaborative networks, innovation and knowledge leaders. Drug Discov Today. (2022) 27:103396. doi: 10.1016/j.drudis.2022.103396 36241041

[2] Ghofrani HA Osterloh IH Grimminger F . Sildenafil: from angina to erectile dysfunction, pulmonary hypertension and beyond. Nat Rev Drug Discov. (2006). 5(8):689–702. doi: 10.1038/nrd2030 16883306 PMC7097805

[3] Amare GG Meharie BG Belayneh YM . A drug repositioning success: The repositioned therapeutic applications and mechanisms of action of thalidomide. J Oncol Pharm Pract. (2021) 27:673–8. doi: 10.1177/1078155220975825 33249990

[4] Kamgar-Dayhoff P Brelidze TI . Multifaceted effect of chlorpromazine in cancer: Implications for cancer treatment. Oncotarget. (2021) 12:1406–26. doi: 10.18632/oncotarget.28010 34262651 PMC8274723

[5] Kapur S Mamo D . Half a century of antipsychotics and still a central role for dopamine D2 receptors. Prog Neuropsychopharmacol Biol Psychiatry. (2003). 27(7):1081–90. doi: 10.1016/j.pnpbp.2003.09.004 14642968

[6] Mortensen PB . Neuroleptic treatment and other factors modifying cancer risk in schizophrenic patients. Acta Psychiatr Scand. (1987) 75:585–90. doi: 10.1111/j.1600-0447.1987.tb02839.x 2887088

[7] Tatsumi M Groshan K Blakely RD Richelson E . Pharmacological profile of antidepressants and related compounds at human monoamine transporters. Eur J Pharmacol. (1997) 340(2-3):249–58. doi: 10.1016/S0014-2999(97)01393-9 9537821

[8] Asensi-Cantó A López-Abellán MD Castillo-Guardiola V Hurtado AM Martínez-Penella M Luengo-Gil . Antitumoral effects of tricyclic antidepressants: Beyond neuropathic pain treatment. Cancers (Basel). (2022) 14:3248. doi: 10.3390/cancers14133248 35805019 PMC9265090

[9] Karawekpanyawong N Kaewkitikul K Maneeton B Maneeton N Siriaree S . The prevalence of depressive disorder and its association in Thai cervical cancer patients. PloS One. (2021) 16:e0252779. doi: 10.1371/journal.pone.0252779 34153051 PMC8216533

[10] Hartung TJ Brähler E Faller H Härter M Hinz A Johansen C . The risk of being depressed is significantly higher in cancer patients than in the general population: Prevalence and severity of depressive symptoms across major cancer types. Eur J Cancer. (2017) 72:46–53. doi: 10.1016/j.ejca.2016.11.017 28024266

[11] Kautio AL Haanpää M Saarto T Kalso E . Amitriptyline in the treatment of chemotherapy-induced neuropathic symptoms. J Pain Symptom Manage. (2008) 35:31–9. doi: 10.1016/j.jpainsymman.2007.02.043 17980550

[12] Sindrup SH Otto M Finnerup NB Jensen TS . Antidepressants in the treatment of neuropathic pain. Basic Clin Pharmacol Toxicol. (2005) 96:399–409. doi: 10.1111/j.1742-7843.2005.pto_96696601.x 15910402

[13] Arokia Femina T Barghavi V Archana K Swethaa NG Maddaly R . Non-uniformity in *in vitro* drug-induced cytotoxicity as evidenced by differences in IC50 values - implications and way forward. J Pharmacol Toxicol Methods. (2023) 119:107238. doi: 10.1016/j.vascn.2022.107238 36521817

[14] Dravid A Raos B Svirskis D O’Carroll SJ . Optimised techniques for high-throughput screening of differentiated SH-SY5Y cells and application for neurite outgrowth assays. Sci Rep. (2021) 11:23935. doi: 10.1038/s41598-021-03442-1 34907283 PMC8671469

[15] Asiri A Tasleem M Al Said M Asiri A Al Qarni AA Bakillah A . Optimizing cell density and unveiling cytotoxic profiles of DMSO and ethanol in six cancer cell lines: Experimental and in silico insights. Methods Protoc. (2025) 8:93. doi: 10.3390/mps8040093 40863743 PMC12388702

[16] Suarez-Arnedo A Torres Figueroa F Clavijo C Arbeláez P Cruz JC Muñoz-Camargo C . An image J plugin for the high throughput image analysis of *in vitro* scratch wound healing assays. PloS One. (2020) 15:e0232565. doi: 10.1371/journal.pone.0232565 32722676 PMC7386569

[17] Dryer Y Berghausen J Creswell K Glasgow E Brelidze TI . Comparison of tumor growth assessment using GFP fluorescence and DiI labeling in a zebrafish xenograft model. Cancer Biol Ther. (2023) 24:2234140. doi: 10.1080/15384047.2023.2234140 37455418 PMC10353338

[18] Roth SM Berens EB Sharif GM Glasgow E Wellstein A . Cancer cell invasion and metastasis in zebrafish models (Danio rerio). Methods Mol Biol. (2021) 2294:3–16. doi: 10.1007/978-1-0716-1350-4_1 33742390 PMC9107928

[19] Hutchinson TH Shillabeer N Winter MJ Pickford DB . Benefits of the maximum tolerated dose (MTD) and maximum tolerated concentration (MTC) concept in aquatic toxicology. Aquat Toxicol. (2009) 91(2):111–6. doi: 10.1016/j.aquatox.2008.11.009 19124163

[20] Berghausen J Thomas CAD Wang ZJ Kihn K Glasgow E Brelidze TI . Amitriptyline inhibits EAG1 channels by binding to their PAS domains and exerts EAG1-dependent anti-tumorigenic effects. Cancer Gene Ther. (2026) 16:1–14. doi: 10.1038/s41417-026-01029-4 41992030 PMC13461354

[21] Dillekås H Rogers MS Straume O . Are 90% of deaths from cancer caused by metastases? Cancer Med. (2019) 8:5574–6. doi: 10.1002/cam4.2474 31397113 PMC6745820

[22] Lee WY Lee WT Cheng CH Chen KC Chou CM Chung CH . Repositioning antipsychotic chlorpromazine for treating colorectal cancer by inhibiting sirtuin 1. Oncotarget. (2015) 6:27580–95. doi: 10.18632/oncotarget.4768 26363315 PMC4695010

[23] Udrea AM Staicu A Smarandache A Andrei IR Badea MA Avram S . Enhancement of chlorpromazine efficacy in breast cancer treatment by 266 nm laser irradiation. Sci Rep. (2024) 14:30329. doi: 10.1038/s41598-024-82088-1 39639119 PMC11621703

[24] Jhou AJ Chang HC Hung CC Lin HC Lee YC Liu WT . Chlorpromazine, an antipsychotic agent, induces G2/M phase arrest and apoptosis via regulation of the PI3K/AKT/mTOR-mediated autophagy pathways in human oral cancer. Biochem Pharmacol. (2021) 184:114403. doi: 10.1016/j.bcp.2020.114403 33388284

[25] Kurita J Hirao Y Nakano H Fukunishi Y Nishimura Y . Sertraline, chlorprothixene, and chlorpromazine characteristically interact with the REST-binding site of the corepressor mSin3, showing medulloblastoma cell growth inhibitory activities. Sci Rep. (2018) 8:13763. doi: 10.1038/s41598-018-31852-1 30213984 PMC6137095

[26] Adornetto A Laganà ML Satriano A Licastro E Corasaniti MT Bagetta G . The antidepressant drug amitriptyline affects human SH-SY5Y neuroblastoma cell proliferation and modulates autophagy. Int J Mol Sci. (2024) 25:10415. doi: 10.3390/ijms251910415 39408742 PMC11476963

[27] Parker KA Glaysher S Hurren J Knight LA McCormick D Suovouri A . The effect of tricyclic antidepressants on cutaneous melanoma cell lines and primary cell cultures. Anticancer Drugs. (2012) 23:65–9. doi: 10.1097/CAD.0b013e32834b1894 21897201

[28] Arimochi H Morita K . Characterization of cytotoxic actions of tricyclic antidepressants on human HT29 colon carcinoma cells. Eur J Pharmacol. (2006) 541:17–23. doi: 10.1016/j.ejphar.2006.04.053 16753142

[29] Wang Y Wang X Wang X Wu D Qi J Zhang Y . Imipramine impedes glioma progression by inhibiting YAP as a Hippo pathway independent manner and synergizes with temozolomide. J Cell Mol Med. (2021) 25:9350–63. doi: 10.1111/jcmm.16874 34469035 PMC8500960

[30] Brodnanova M Hatokova Z Evinova A Cibulka M Racay P . Differential impact of imipramine on thapsigargin- and tunicamycin-induced endoplasmic reticulum stress and mitochondrial dysfunction in neuroblastoma SH-SY5Y cells. Eur J Pharmacol. (2021) 902:174073. doi: 10.1016/j.ejphar.2021.174073 33798597

[31] Xu F Xi H Liao M Zhang Y Ma H Wu M . Repurposed antipsychotic chlorpromazine inhibits colorectal cancer and pulmonary metastasis by inducing G2/M cell cycle arrest, apoptosis, and autophagy. Cancer Chemother Pharmacol. (2022) 89:331–46. doi: 10.1007/s00280-021-04386-z 35067737

[32] Eisenberg S Giehl K Henis YI Ehrlich M . Differential interference of chlorpromazine with the membrane interactions of oncogenic K-Ras and its effects on cell growth. J Biol Chem. (2008) 283:27279–88. doi: 10.1074/jbc.M804589200 18693247

[33] Lim EY Park J Kim YT Kim MJ . Imipramine inhibits migration and invasion in metastatic castration-resistant prostate cancer PC-3 cells via AKT-mediated NF-κB signaling pathway. Molecules. (2020) 25:4619. doi: 10.3390/molecules25204619 33050597 PMC7587212

[34] Wang W Liu Y Lo T Hsu F Chiang C . Imipramine‐induced apoptosis and metastasis inhibition in human bladder cancer T24 cells through EGFR/ERK/NF‐ĸB pathway suppression. In Vivo. (2025) 39:669–82. doi: 10.21873/invivo.13871 40010952 PMC11884455

[35] Timilsina S Rajamanickam S Rao A Subbarayalu P Nirzhor S Abdelfattah N . The antidepressant imipramine inhibits breast cancer growth by targeting estrogen receptor signaling and DNA repair events. Cancer Lett. (2022) 540:215717. doi: 10.1016/j.canlet.2022.215717 35568265 PMC10313451

[36] Vandel S Vandel B Sandoz M Allers G Bechtel P Volmat R . Clinical response and plasma concentration of amitriptyline and its metabolite nortriptyline. Eur J Clin Pharmacol. (1978) 14:185–90. doi: 10.1007/BF02089958 365538

[37] Ulrich S Läuter J . Comprehensive survey of the relationship between serum concentration and therapeutic effect of amitriptyline in depression. Clin Pharmacokinet. (2002) 41:853–76. doi: 10.2165/00003088-200241110-00004 12190332

[38] Curry SH Marshall JH Davis JM Janowsky DS . Chlorpromazine plasma levels and effects. Arch Gen Psychiatry. (1970) 22:289–96. doi: 10.1001/archpsyc.1970.01740280001001 5417623

[39] Cooper TB Simpson GM Lee JH . Thymoleptic and neuroleptic drug plasma levels in psychiatry: Current status. Int Rev Neurobiol. (1976) 19:269–309. doi: 10.1016/s0074-7742(08)60706-0 1010700

[40] Sindrup SH Gram LF Skjold T Frøland A Beck-Nielsen H . Concentration-response relationship in imipramine treatment of diabetic neuropathy symptoms. Clin Pharmacol Ther. (1990) 47:509–15. doi: 10.1038/clpt.1990.65 2328559

[41] Baldessarini RJ Centorrino F Flood JG Volpicelli SA Huston-Lyons D Cohen BM . Tissue concentrations of clozapine and its metabolites in the rat. Neuropsychopharmacology. (1993) 9:117–24. doi: 10.1038/npp.1993.50 8216694

[42] Kornhuber J Schultz A Wiltfang J Meineke I Gleiter CH Zöchling R . Persistence of haloperidol in human brain tissue. Am J Psychiatry. (1999) 156:885–90. doi: 10.1176/ajp.156.6.885 10360127

[43] Seeman P . Anti-schizophrenic drugs--membrane receptor sites of action. Biochem Pharmacol. (1977) 26:1741–8. doi: 10.1016/0006-2952(77)90340-9 20896

[44] Csatary LK . Chlorpromazines and cancer. Lancet. (1972) 2(7772):338–9. doi: 10.1016/s0140-6736(72)92955-8 4115079

[45] Mortensen PB . Neuroleptic medication and reduced risk of prostate cancer in schizophrenic patients. Acta Psychiatrica Scandinavica. (1992) 85(5):390–3. doi: 10.1111/j.1600-0447.1992.tb10325.x 1351334

[46] Chung CM Kuo TM Chiang SL Wang ZH Hung CC Lane HY . Antidepressants in association with reducing risk of oral cancer occurrence: A nationwide population-based cohort and nested case-control studies. Oncotarget. (2016) 7:11687–95. doi: 10.18632/oncotarget.7049 26840257 PMC4905503

[47] Walker AJ Card T Bates TE Muir K . Tricyclic antidepressants and the incidence of certain cancers: A study using the GPRD. Br J Cancer. (2011) 104:193–7. doi: 10.1038/sj.bjc.6605996 21081933 PMC3039809

[48] Wang CH Huang CW Nguyen NTH Lin MC Nguyen PA Islam MM . Real-world data on the associations of tricyclic antidepressants and selective serotonin reuptake inhibitors with gynecologic cancer risk. Cancers (Basel). (2025) 17:1616. doi: 10.3390/cancers17101616 40427115 PMC12109922

[49] Sharpe CR Collet JP Belzile E Hanley JA Boivin JF . The effects of tricyclic antidepressants on breast cancer risk. Br J Cancer. (2002) 86:92–7. doi: 10.1038/sj.bjc.6600013 11857018 PMC2746543

[50] Ma Y He J Li C Liu F Wang Y Song F . Effect of antidepressants use on cancer morbidity and mortality: A propensity score-matched longitudinal cohort study. J Affect Disord. (2025) 387:119554. doi: 10.1016/j.jad.2025.119554 40449745

