# Supplementary figures and images for: Repurposing tricyclic drugs as cancer therapeutics: comparative analysis of antitumorigenic effects of chlorpromazine, amitriptyline and imipramine
Source: Front Oncol. 2026 Jul 2;16:1827698. doi: 10.3389/fonc.2026.1827698 (PMC13372609; doi:10.3389/fonc.2026.1827698)

A

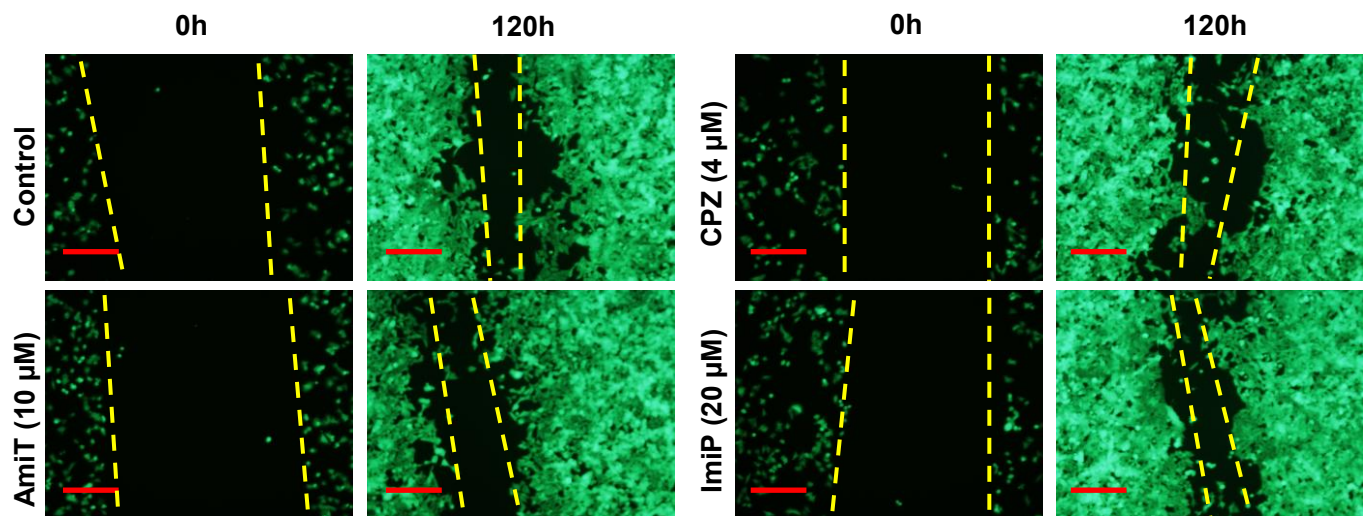

B

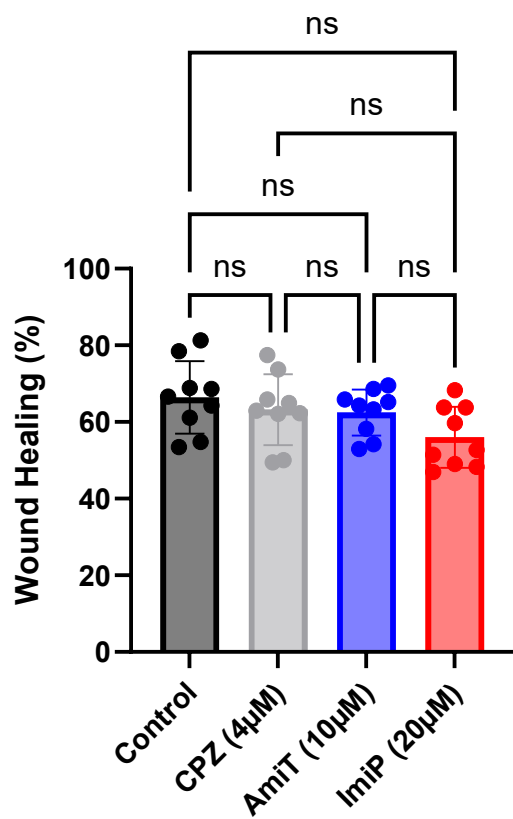

Supplement: Supplementary Figure 1 — CPZ, AmiT, and ImiP do not affect cell migration in A375 cells. (A) Representative images of the wound healing of A375 cells, untreated or treated with 4 µM CPZ, 10 µM AmiT or 20 µM at 0 and 120 hours after scratch. Scale bar = 0.25 mm. (B) Bar graph summary of the % wound healing in the absence (black) and presence of 4 µM CPZ (grey), 10 µM AmiT (blue) or 20 µM (red) at 120 hours post-scratch for A375 cells. Statistical significance was determined with ANOVA test (ns P > 0.05). The data are presented as mean ± SD and are averages of three technical replicates for three different biological samples for each tested condition. [file DataSheet1.pdf]
